# Sanguinarine Attenuates Collagen-Induced Platelet Activation and Thrombus Formation

**DOI:** 10.3390/biomedicines9050444

**Published:** 2021-04-21

**Authors:** Dan Shu, Ying Zhu, Meng Lu, Ao-Di He, Jiang-Bin Chen, Ding-Song Ye, Yue Liu, Xiang-Bin Zeng, Rong Ma, Zhang-Yin Ming

**Affiliations:** 1 Department of Pharmacology, School of Basic Medicine, Tongji Medical College of Huazhong, University of Science and Technology, 13 Hangkong Road, Wuhan 430030, China; d201781151@hust.edu.cn (D.S.); d201881246@hust.edu.cn (Y.Z.); d201981341@hust.edu.cn (M.L.); u201610406@hust.edu.cn (J.-B.C.); u201610404@hust.edu.cn (D.-S.Y.); m201875087@hust.edu.cn (Y.L.); m201975292@hust.edu.cn (X.-B.Z.); marong@hust.edu.cn (R.M.); 2 The Key Laboratory for Drug Target Research and Pharmacodynamic Evaluation of Hubei Province, 13 Hangkong Road, Wuhan 430030, China; 3 College of Pharmacy, Xiangnan University, 889 Chenzhou Avenue, Chenzhou 423000, China; 4 Department of Physiology, School of Basic Medicine, Huazhong University of Science and Technology, Wuhan 430030, China; adhe@hust.edu.cn; 5 Wuhan Center for Brain Science, Huazhong University of Science and Technology, Wuhan 430030, China; 6 Tongji-Rongcheng Center for Biomedicine, Huazhong University of Science and Technology, Wuhan 430030, China

**Keywords:** sanguinarine, antithrombotic, platelet, glycoprotein VI pathway, integrin αIIbβ3

## Abstract

Sanguinarine, a benzophenanthridine alkaloid, has been described to have an antiplatelet activity. However, its antithrombotic effect and the mechanism of platelet inhibition have not thoroughly been explored. The current study found that sanguinarine had an inhibitory effect on thrombus formation. This inhibitory effect was quite evident both in the flow-chamber assays as well as in a murine model of FeCl_3_-induced carotid artery thrombosis. Further investigations also revealed that sanguinarine inhibited the collagen-induced human platelet aggregation and granule release. At the same time, it also prevented platelet spreading and adhesion to immobilized fibrinogen. The molecular mechanisms of its antiplatelet activity were found to be as follows: 1. Reduced phosphorylation of the downstream signaling pathways in collagen specific receptor GPVI (Syk-PLCγ2 and PI3K-Akt-GSK3β); 2. Inhibition of collagen-induced increase in the intracellular Ca^2+^ concentration ([Ca^2+^]i); 3. Inhibition of integrin αIIbβ3 outside-in signaling via reducing β3 and Src (Tyr-416) phosphorylation. It can be concluded that sanguinarine inhibits collagen-induced platelet activation and reduces thrombus formation. This effect is mediated via inhibiting the phosphorylation of multiple components in the GPVI signaling pathway. Current data also indicate that sanguinarine can be of some clinical value to treat cardiovascular diseases involving an excess of platelet activation.

## 1. Introduction

Platelets are discoid, anucleate cells that are continuously produced by megakaryocytes. They are key factors during hemostasis and thrombosis, along with other physiological and pathological processes [1]. Epidemiological investigations indicate that several risk factors of thrombosis are related to high platelet activity and excessive activation of endothelial cell procoagulant functions. Moreover, abnormal platelet adhesion, aggregation, granule release and spreading can be the basis of thrombotic disease [2]. The subendothelial matrix proteins collagen and von Willebrand factor (vWF) are exposed by the damages to endothelium. Thrombus is initiated by adhesion of platelet to collagen (via GPVI) and vWF (via GPIb-V-IX) [1]. Syk-PLCγ2 and PI3K-Akt signaling pathways are involved in collagen-activated platelets. Then it results in mobilization of cytoplasmic calcium, platelet shape change and granule release [3,4,5]. Platelets release a few soluble agonists, including ADP and thromboxane A2. Thus, intensifying activated platelets attracts more platelets from circulation to enlarge the thrombus [6]. Arterial thrombosis from platelet activation leads to the acute severe symptoms of heart disease and thrombotic stroke [7]. Currently, the most effective and clinically recommended antiplatelet drugs are cyclooxygenase-1, platelet adenosine diphosphate (ADP) receptor P2Y_12_ and integrin αIIbβ3 inhibitors [8]. However, all the recommended treatments do have a few limitations, specifically in single-target treatments. These limitations lead to drug resistance and poor selectivity, which in turn increase the risk of irreversible bleeding [9]. In recent years, more researchers have focused on the antiplatelet and antithrombotic activities of natural compounds in Chinese herbal medicine [10].

Sanguinarine, a benzophenanthridine alkaloid, is found in a variety of plants, including *Macleaya cordata*, *Herba sanguinariae* and *Lysimachia platyphylla* [11]. As an FDA-approved compound, it is used as a health product to inhibit the growth of dental plaque [12]. Other biological and pharmacological activities of sanguinarine include anti-inflammatory, antioxidant and antitumor. Recently, these activities have attracted greater attention from researchers around the world [13]. A study by Jeng reported that the effect of sanguinarine on platelet activation in vitro is related to the production of thromboxane and cAMP [14]. However, the role of sanguinarine in platelet inhibition and thrombosis is not completely elucidated to date. Here, we report that sanguinarine attenuates collagen-induced platelet activation and thrombus formation by targeting the GPVI pathway, intracellular Ca^2+^ mobilization and integrin αIIbβ3-mediated “outside-in” signal transduction.

## 2. Materials and Methods

### 2.1. Chemicals and Reagents

**Figure 1:**
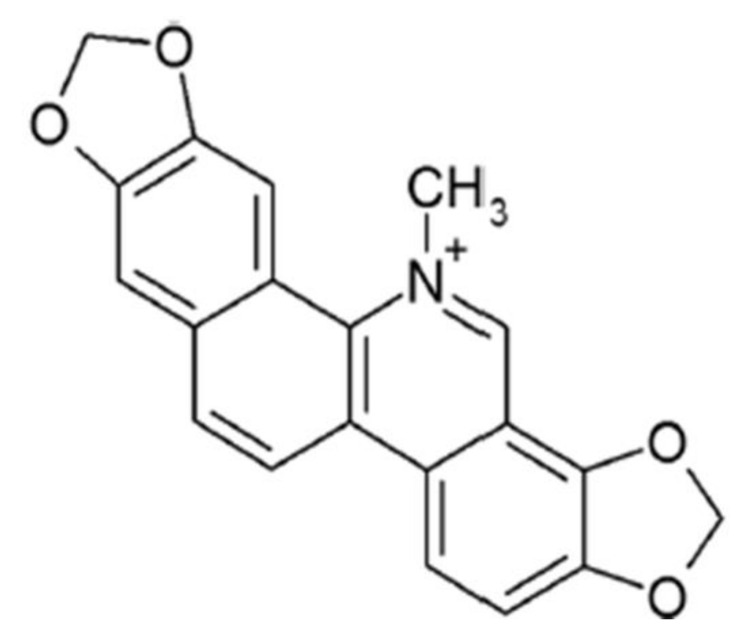
Chemical structure of sanguinarine.

Sanguinarine (13-methyl(1,3) benzodioxolo (5,6-c) -1,3-dioxolo(4,5-i) phenanthridinium), (HPLC ≥ 98%, SA, Figure 1) (Absin, Shanghai, China), was dissolved in 0.1% DMSO (final concentration). The EDTA, prostaglandin E1(PGE1) and bovine serum albumin (BSA) were obtained from Sigma (St. Louis, MO, USA). The CHRONO-LUME reagent and collagen were acquired from Chrono-Log Corp. (Havertown, PA, USA). The collagen-related peptide was obtained from Dr. Newman’s lab (Blood Center of Wisconsin, Milwaukee, WI, USA). The FITC-conjugated anti-CD62P (P-selectin) antibody and FITC-conjugated anti-PAC-1(αIIbβ3) antibody were purchased from Biolegend (San Diego, CA, USA). The Fluo-3AM was purchased from MCE (Shanghai, China). The CellTrace Calcein Green was purchased from Invitrogen (Carlsbad, CA, USA). The anti-phospho-PI3K(Ser1070), anti-PI3K, anti-phospho-Akt(Ser473), anti-phospho-GSK3β (Ser9), anti-phoshpo-PLCγ2 (Tyr1217), anti-β3, anti-phospho-β3 (Tyr474), anti-Src, anti-phospho-Src (Tyr416) and anti-phospho-Syk (Tyr525/526) antibodies were purchased from Cell Signaling Technology (Beverly, MA, USA). The anti-Syk, anti-PLCγ2, anti-Akt and antil-GSK3β were acquired from Santa Cruz Biotechnology (Dallas, TX, USA).

### 2.2. FeCl_3_-Induced Carotid Arterial Injury Thrombosis Model

To determine the antithrombotic effect of sanguinarine, we measured the occlusion time in the FeCl_3_-induced carotid arterial injury thrombosis model, which has been shown to cause substantial endothelium damage with exposure of underlying collagens [15]. The protocol was adapted from previously published work with minor changes [16]. C57BL/6 mice (8–12 weeks old) were obtained from Hubei Experimental Animal Research Center (Wuhan, Hubei, China). The mice experiments were evaluated and permitted by the Ethics Committee for experimental animals, Tongji Medical College. Sanguinarine (5 mg/kg or 10 mg/kg) was given intragastrically 24 h, 12 h and 2 h before modeling. The mice were given anesthetics by intraperitoneal injection of pentobarbital sodium before the surgical operations in which their carotid arteries were exposed. A Doppler flow probe (Transonic, TS420, UK) was used to measure the baseline blood flow in the proximal carotid artery. A 2 mm strip of filter paper saturated with 10% ferric chloride was introduced into the carotid artery adventitial surface for 1 min. Then the filter paper was removed, and blood flow through the carotid artery was then observed carefully for 45 min or until 95% vessel occlusion was achieved.

### 2.3. Bleeding-Time Tests

The bleeding time of the mouse tails was measured as described previously [17]. Sanguinarine (5 mg/kg or 10 mg/kg) was given intragastrically 24 h, 12 h, and 2 h prior to the surgery. Tails were cut 5 mm from the tip with a razor blade, then the tails were immediately immersed in 37 °C saline, and the time to cessation of bleeding was recorded. A stable cessation of bleeding was established when there was no rebleeding for more than 1 min.

### 2.4. Human Platelet Preparation

The human platelets were isolated from freshly drawn venous blood, collected from 15 healthy volunteers (8 males and 7 females, 18–25 years old) who had not taken any drugs that could affect platelet function in the previous 2 weeks. The study design was approved by the relevant ethics committees. The washed platelets were arranged and organized as reported previously [4,5]. In brief, human blood was drawn from the cubital vein without stasis into siliconized vacutainers that contained 1:9 (*v*/*v*) 3.8% trisodium citrate. Platelets were rinsed with Tyrode’s buffer (137 mM NaCl, 13.8 mM NaHCO_3_, 5.5 mM glucose, 2.5 mM KCl, 20 mM HEPES, 0.36 mM NaH_2_PO_4_, pH 7.4) containing 1 μM PGE1 and 2.5 mM EDTA, and then placed in suspension in Tyrode’s buffer not containing PGE1 or EDTA at a final concentration of 3 × 10^8^/mL. All preparations for human platelets were conducted at room temperature. Platelets that were resuspended needed to rest for at least 30 min before experiment.

### 2.5. Platelet Aggregation and ATP Release Assay

Platelet aggregation and ATP release assays were executed according to the previous reports [5]. The platelet pellet was resuspended in Tyrode’s buffer, and the concentration was then adjusted to 3 × 10^8^/mL for the aggregation studies. Later, the platelets were preincubated with either sanguinarine alone or DMSO (control) for 10 min at 37 °C. Prior to individual agonist stimulations, CaCl_2_ (1 mM) was added. Using a luciferin–luciferase reagent (CHRONO-LUME, Chrono-Log, Havertown, PA, USA), the release of ATP was measured. The ATP release was documented and examined with the Aggregolink software (Chrono-Log, Havertown, PA, USA).

### 2.6. Flow Cytometric Analysis

Washed human platelets were preincubated with sanguinarine (1, 2.5 and 5 μM) or DMSO (control) for 10 min before incubating with collagen (2 μg/mL) at 37 °C for 5 min. The binding of FITC-conjugated anti-CD62P (P-selectin) antibody and FITC-conjugated anti-PAC-1 (αIIbβ3) antibody to platelets (5 × 10^7^/mL) was performed by incubation in the dark at room temperature for 15 min, and was later evaluated with a BD Biosciences flow cytometer (San Jose, CA, USA).

### 2.7. Platelet Adhesion on the Collagen-Coated Surface under Flow

The experiments were performed as described previously with minor modifications [18]. The Bioflux plates (Bioflux 1000Z, Fluxion Biosciences Inc., South San Francisco, CA, USA) were covered with 100 µg/mL collagen overnight at 4 °C. Washed human platelets were labeled with CellTrace Calcein Green (0.34 µM, at 37 °C for 30 min) and then incubated with DMSO (control) or sanguinarine (1, 2.5 and 5 μM) for 10 min. The mixture of labeled platelets and washed red blood cells (with a hematocrit of about 40%) and CaCl_2_/MgCl_2_ (75 mM/37.5 mM) was perfused through Bioflux plates at a wall shear rate of 2000 s^−1^ for 5 min. To detect platelet adhesion, a 10× long-working-distance objective was utilized for fluorescence and transmitted light microscopy. The formation of thrombus was detailed using an inverted epifluorescence microscope (Zeiss Microscope, Shanghai, China) integrated with the software. The platelet coverage was then evaluated based on the intensity and coverage area with the Bioflux Montage software (Fluxion Biosciences Inc., South San Francisco, CA, USA).

### 2.8. Calcium Signaling

Intracellular Ca^2+^ flux was measured by flow cytometry [19]. Washed human platelets (5 × 10^7^/mL) were labeled with 1 μM Fluo-3AM at 37 °C for 30 min, then incubated with sanguinarine (1, 2.5 and 5 μM) or DMSO (control) for 10 min. After collecting a baseline reading for 20 s, collagen (2 µg/mL) and 2 mM extracellular calcium were added to the FACS tube, and the change in fluorescence intensity was recorded on a BD Biosciences flow cytometer (BD Biosciences, Franklin Lakes, NJ, USA) and analyzed using Flowjo V10 (BD Biosciences, Franklin Lakes, NJ, USA). The fitting curve was plotted using SigmaPlot software 14.0 (Systat Software, Inc., San Jose, CA, USA).

### 2.9. Platelet Spreading on Immobilized Fibrinogen

Platelet spreading on the surfaces covered with fibrinogen was conducted as described previously [20]. Platelet adhesion was observed using a fluorescence microscope (OLYMPUS BX51, Olympus (China) Co., Ltd., Beijing, China), images were obtained using an OLYMPUS CCD camera, then the spreading area of individual platelets was analyzed with Image J software 1.8.0 (NIH open source, Bethesda, MD, USA).

### 2.10. Immunoblot Analysis

For the detection of phosphorylated proteins, aggregated or spreading platelets underwent lysis by adding an equal volume of 2× lysis buffer (30 mM HEPES, 300 mM NaCl, 20 mM EGTA, 0.2 mM MgCl_2_, 2% Triton X-100, 2× protease, 2× phosphatase inhibitor cocktails) into the reactions. The lysates were separated by SDS-PAGE (8% Bis Tris Gel) and transferred onto a PVDF membrane (Invitrogen). Then the membrane was incubated with the following antibodies: pSyk (Tyr525/526), Syk, pPLCγ2 (Tyr1217), PLCγ2, pPI3K(Ser1070), PI3K, pAkt(Ser473), Akt, pGSK3β (Ser9), GSK3β, pβ3 (Tyr474), β3, pSrc (Tyr416), Src. GAPDH served as the internal standard. Protein bands were visualized by an enhanced chemiluminescence assay kit (Thermo Fisher Scientific (China) Co., Ltd., Shanghai, China). Signal quantification was performed on raw images using Image J software 1.8.0 (NIH open source, Bethesda, MD, USA).

### 2.11. Statistical Analysis

Data were analyzed using Graph Pad Software 7.0 and the results were expressed as the means ± SEM, while the variance of the data was also used to evaluate the data. A two-tailed unpaired comparison test using a t-test was adopted to analyze the difference between two groups. One-way analysis of covariance (ANOVA) was utilized to compare more than two groups. *p* < 0.05 was considered significant.

## 3. Results

### 3.1. Sanguinarine Inhibits Arterial Thrombosis In Vivo and In Vitro

**Figure 2:**
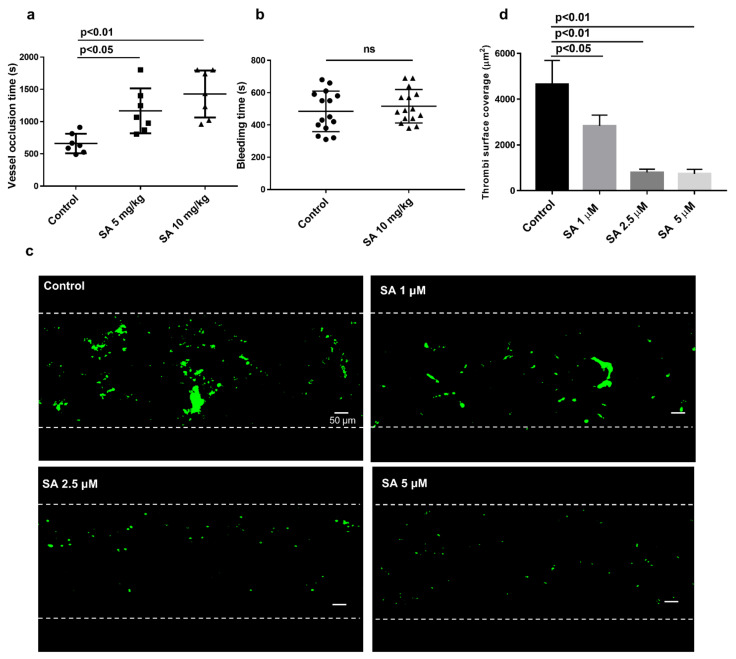
Sanguinarine inhibits the formation of thrombus in vivo and in vitro without prolonging bleeding time. (**a**) Summary data of ferric chloride (FeCl_3_)-induced thrombus formation. Thrombus formation in vivo was assessed by occlusion time(s) after FeCl_3_ -induced injury of the carotid artery, which was measured with a doppler flow probe. The occlusion time of the sanguinarine-treated (5 mg/kg or 10 mg/kg, i.g.) were compared with the control (saline + 5% DMSO i.g.) group. The results were expressed as mean ± SEM (*n* = 7) and were analyzed using one-way ANOVA. (**b**) The effect of sanguinarine on bleeding time. Results are shown as mean ± SEM (*n* = 15), and data were analyzed using an unpaired t test. (**c**) Sanguinarine reduced thrombosis in vitro. Washed human platelets were labeled with CellTrace Calcein Green and then incubated with DMSO (control) or sanguinarine (1, 2.5 and 5 μM) for 10 min. The mixture of labeled platelets and washed red blood cells (with a hematocrit of about 40%) and CaCl_2_/MgCl_2_ (75 mM/37.5 mM) was perfused through Bioflux plates at a wall shear rate of 2000 s^−1^ for 5 min. Representative images of surface coverage were revealed. (**d**) Summary data of area covered by platelets at the 5 min time point were studied and measured (scale bars, 50 μM). Results and data were collected from at least five separate independent experiments, analyzed using one-way ANOVA analysis and presented as mean ± SEM (*n* = 5).

To check sanguinarine antithrombotic effect in vivo, we used the FeCl_3_ injury-induced carotid thrombosis model. A doppler flow meter detected the vessel occlusion time. While sanguinarine has great liver toxicity by intravenous and intraperitoneal injection, it is relatively safe for intragastric administration. The LD_50_ of sanguinarine is less than 200 mg/kg by intragastric administration [21]. Its T_MAX_ is 2 h, and it completely eliminated from the plasma and liver after 24 h [11]. Sanguinarine (5 mg/kg or 10 mg/kg) was given intragastrically 24 h, 12 h and 2 h before modeling. The results indicated that the average occlusion times in 5 mg/kg and 10 mg/kg groups were 1166 s and 1427 s, respectively, which were much longer than that in the control group (660 s) (both *p* < 0.05) (Figure 2a). Nevertheless, the tail bleeding time was not prolonged in the sanguinarine treatment group (Figure 2b). This implies that the drug did not cause bleeding at the antithrombotic dose. Under normal and pathological conditions in vivo, the shear force exerted by blood laminar flow is considered an important environmental factor during platelet activation and thrombosis. [22]. To further demonstrate sanguinarine antithrombotic effect in clinic, a microfluidic system (Bioflux 1000Z, Fluxion Biosciences Inc., South San Francisco, CA, USA) was used to simulate the adhesion of human platelets to collagen matrix under high shear stress of arterial stenosis in vivo. We found that 1 μM, 2.5 μM and 5 μM sanguinarine significantly reduced the adhesion area of platelets to the collagen matrix under arterial shear (Figure 2c,d).

### 3.2. Sanguinarine Inhibits Human Platelet Aggregation Induced by Collagen and Collagen-Related Peptide

**Figure 3:**
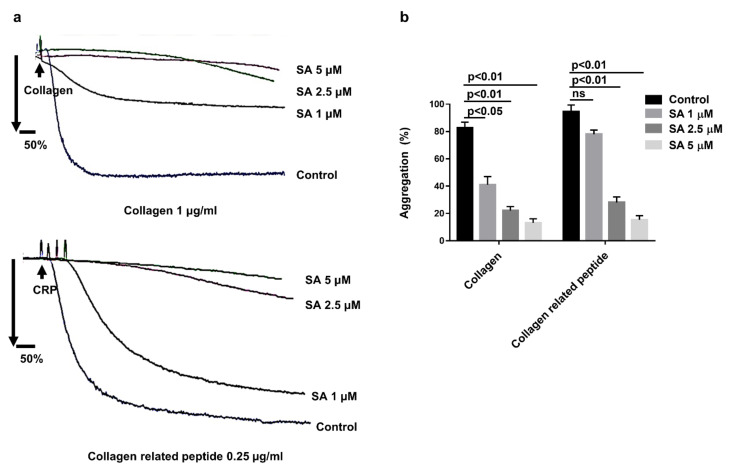
Effects of sanguinarine on collagen and collagen-related peptide-induced human platelet aggregation of washed human platelets. (**a**) Washed human platelets (3 × 10^8^/mL) were preincubated for 10 min with different concentrations of sanguinarine (1, 2.5, 5 µM) or DMSO (control), followed by stimulation with different agonists. (**b**) Data were shown as mean ± SEM (*n* = 5) and examined using one-way ANOVA analysis.

As the arterial injury initially exposed the collagen under the endothelium, the current study focused on checking sanguinarine’s effect on collagen-induced human platelet aggregation. Platelet aggregation was induced by collagen (1 μg/mL) and CRP (0.25 μg/mL) after different doses of sanguinarine or DMSO (control) pretreated at 37 °C for 10 min. The results indicated that sanguinarine inhibited platelet aggregation induced by collagen and collagen-related peptide in a dose-dependent manner. The high dose (5 μM) sanguinarine almost completely inhibited collagen and collagen-related peptide-induced platelet aggregation (Figure 3b).

### 3.3. Sanguinarine Inhibits Collagen-Induced Human Platelet Granule Secretion and Integrin αIibβ3 Activation

**Figure 4:**
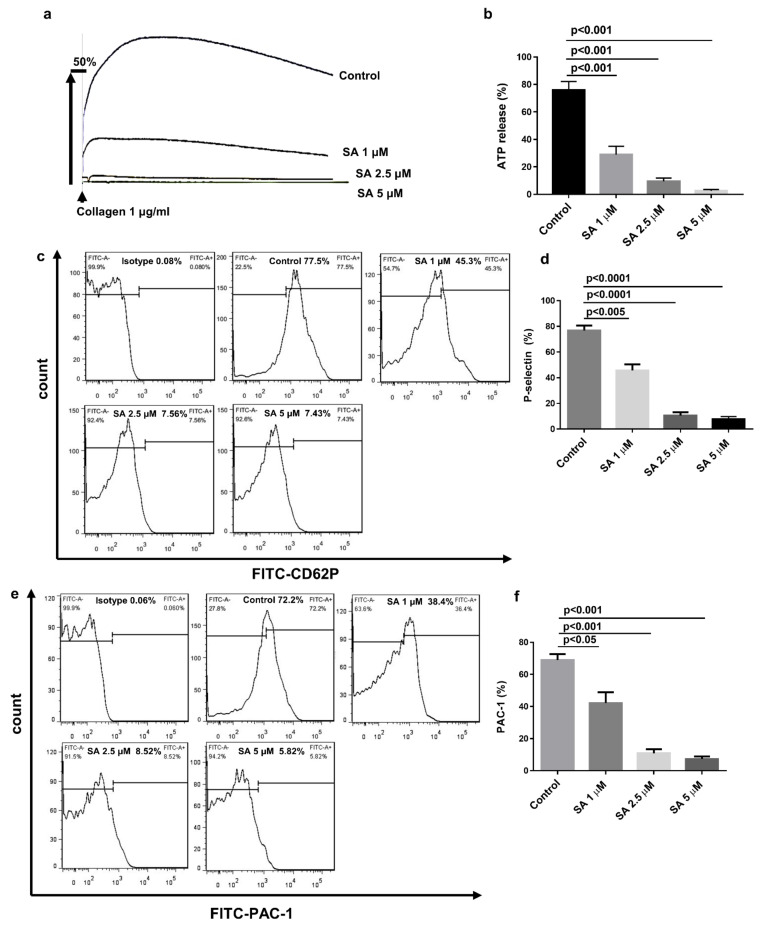
Sanguinarine decreases collagen-induced human platelet granule release and integrin αIIbβ3 activation. (**a**) Washed human platelets were prepared as shown in Figure 3. ATP secretion was documented and computed following the instructions in the Materials and Methods Section. (**b**) Data were shown as mean ± SEM (*n* = 5) and examined using one-way ANOVA analysis. (**c**–**f**) First, washed human platelets (5 × 10^7^/mL) were preincubated for 10 min at 37 °C with sanguinarine (1, 2.5, 5 µM) or DMSO (control) in the presence of FITC-conjugated P-selectin and FITC-conjugated PAC-1 antibodies. Afterward, the platelets were stimulated with collagen (2 µg/mL) and incubated for another 5 min. P-selectin expression (**c**,**d**) and PAC-1 binding (**e**,**f**) were identified by flow cytometry. Results and data were expressed as mean ± SEM (*n* = 5).

To examine the influence of sanguinarine on platelet granule release, human platelets were initially incubated with various doses of sanguinarine at 37 °C for 10 min, and then stimulated with collagen. As shown in Figure 4a,b, sanguinarine reduced collagen-induced platelet ATP release in a dose-dependent manner. A 5 μM dose of sanguinarine almost completely inhibited the ATP release. In addition, sanguinarine dose-dependently decreased the P-selectin expression and PAC-1 binding. The P-selectin expression decreased from 74.5% to 49.23% (1 μM), 15.09% (2.5 μM) and 14.58% (5 μM), respectively (Figure 4c,d). The PAC-1 binding in collagen-stimulated platelets dropped from 73.67% to 40.63% (1 μM), 8.33% (2.5 μM) and 3.9% (5 μM), respectively (Figure 4e,f). This clearly indicates that sanguinarine had an inhibitory effect on collagen-stimulated P-selectin expression and PAC-1 binding in a dose-dependent manner.

### 3.4. Sanguinarine Inhibits Collagen-Induced Human Platelet Intracellular Signal Transduction

**Figure 5:**
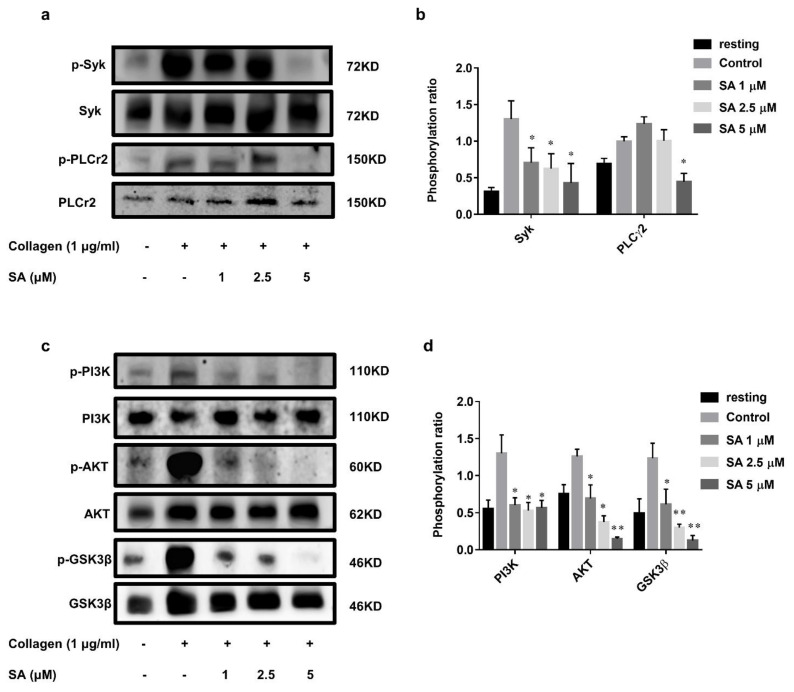
Sanguinarine has an inhibitory effect on collagen-induced phosphorylation of the signaling cascade. (**a**,**c**) Washed human platelets were treated initially with sanguinarine (1, 2.5, 5 µM) or DMSO (control) at 37 °C for 10 min, then stimulated with collagen (1 µg/mL) for 5 min, and later underwent lysis with lysis buffer, followed by Western blotting using specific antibodies. (**b**,**d**) The Syk/PLCγ2 and PI3K/Akt/GSK3β phosphorylation ratios were measured. The blot was a representative of three independent experiments that showed statistical significance at * *p* < 0.05 and ** *p* < 0.01 compared with the control group (*n* = 5).

To further investigate the specific molecular mechanism of sanguinarine in inhibiting collagen-induced human platelet activation, we checked the phosphorylation of several proteins downstream of the GPVI signaling pathway, such as Syk-PLCγ2 and PI3K-Akt-GSK3β. The results (Figure 5a,b) showed that 1 μM, 2.5 μM and 5 μM of sanguinarine significantly inhibited Syk phosphorylation, while only 5 μM of sanguinarine appeared to inhibit PLCγ2 phosphorylation. The PI3K-Akt-GSK3β pathway plays a key role in activating platelets, and is an important component downstream of GPVI signaling [23,24]. As shown in Figure 5c,d, sanguinarine effectively inhibited the phosphorylation of PI3K, Akt and GSK3β in collagen-stimulated platelets. The results suggested that sanguinarine negatively regulated the phosphorylation of Syk-PLCγ2 and PI3K-Akt-GSK3β downstream of platelet GPVI.

### 3.5. Sanguinarine Reduces Platelet Intracellular Ca^2+^ Concentrations ([Ca^2+^]i) in a Dose-Dependent Manner

**Figure 6:**
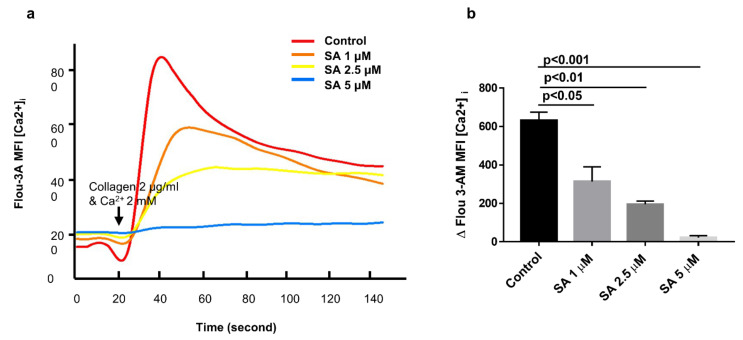
Sanguinarine dose-dependently attenuates intracellular Ca^2+^ concentration ([Ca^2+^]i) in human platelets. (**a**) Intracellular Ca^2+^ flux was measured by flow cytometry. Washed human platelets (5  ×  10^7^/mL) were labeled with 1 μM Fluo-3AM at 37  °C for 30 min, then incubated with sanguinarine (1, 2.5 and 5 μM) or DMSO (control) for 10 min. After collecting a baseline for 20 s, collagen (2 µg/mL) and 2 mM extracellular calcium were added to the FACS tube, and the change in fluorescence intensity was recorded on BD Biosciences flow cytometer and analyzed using Flowjo software. The fitting curve was plotted using Sigma Plot software. (**b**) Values of [Ca^2+^]i elevation upon collagen stimulation shown in (**a**) were plotted as mean ± SEM from five independent experiments.

During the process of platelet activation, intracellular calcium signal plays a very important role [22]. Most of the agonists cause an increase of intracellular calcium concentration after platelet activation [25]. Flou-3AM was used in combination with intracellular free calcium ions to detect the fluorescence intensity by flow cytometry. The extracellular free calcium was complexed with EDTA, and then the extracellular 2 mM calcium and collagen (2 μg/mL) were added to detect the intracellular Ca^2+^ mobilization in platelets. As shown in Figure 6a,b, sanguinarine inhibited the increases in [Ca^2+^]i caused by collagen in a dose-dependent manner, and 5 μM of sanguinarine almost blocked calcium mobilization.

### 3.6. Sanguinarine Negatively Regulates the Integrin αIIbβ3 “Outside-In” Signaling

**Figure 7:**
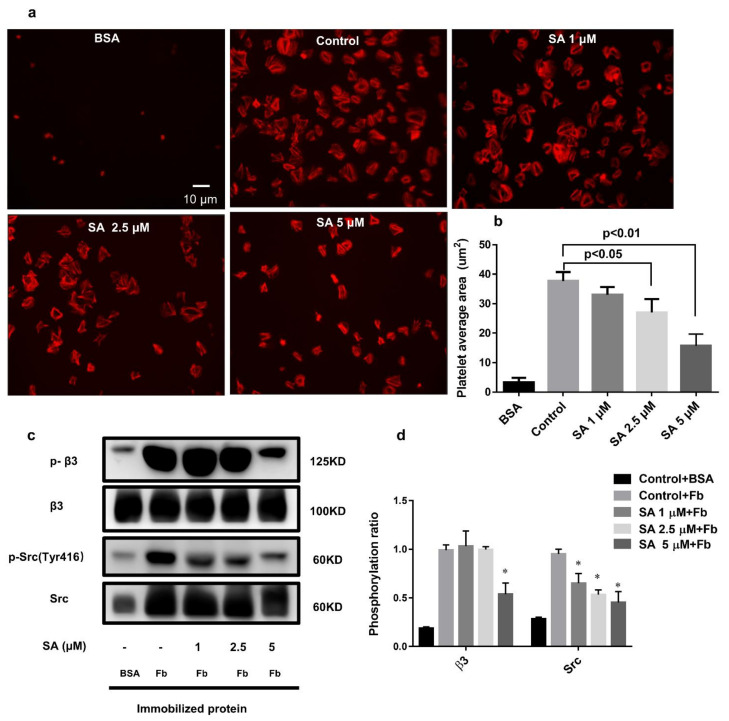
Sanguinarine dose-dependently represses human platelet spreading on the fibrinogen-coated surface and inhibits integrin αIIbβ3-mediated outside-in signaling. (**a**) A representative image of platelets spreading on the coated BSA or fibrinogen cover glasses. (**b**) The figure expressed the quantity of the single adherent platelet area as shown in the right panel, and the average coverage of the spreading platelets in five independent experiments. (**c**,**d**) Typical figure of phosphorylation of β3/Src of lysed spreading platelets for five independent experiments. The data had statistical significance at * *p* < 0.05 when compared to the control group.

We also found that sanguinarine inhibited human platelet adhesion and spreading on the fibrinogen-coated matrix (Figure 7a,b). Compared with the control group, sanguinarine inhibited platelet spreading on immobilized fibrinogen. The platelet surface coverage was much smaller at 5 μM. We further analyzed the molecular mechanism of sanguinarine on platelet outside-in signal transduction by detecting the related protein’s phosphorylationin-spreading platelet. β3 phosphorylation is a direct indicator of αIIbβ3 activation, and Src activation is also associated with outside-in signal transduction [26]. The results demonstrated that phosphorylation of β3 and Src (Tyr 416) was reduced significantly in the presence of sanguinarine (Figure 7c,d). This suggests that sanguinarine plays a significant role in inhibiting the αIIbβ3 outside-in signal transduction.

## 4. Discussion

**Figure 8:**
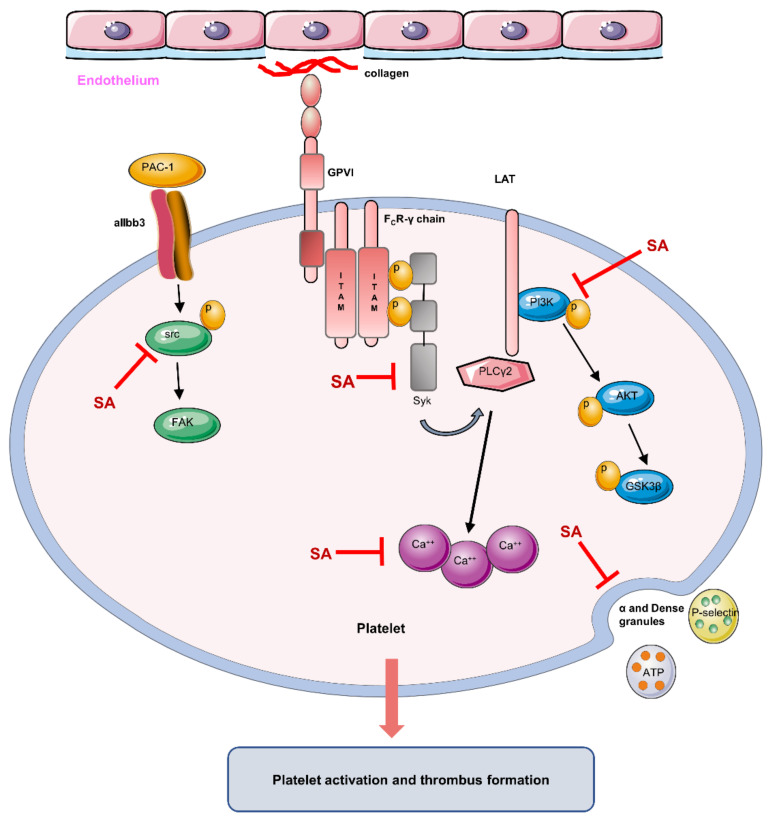
Schematic of sanguinarine antiplatelet activity. Sanguinarine attenuates collagen-induced platelet activation (including α and dense granule release) and thrombus formation by targeting the GPVI pathway (inhibiting the phosphorylation of Syk-PLCγ2 and PI3K-Akt-GSK3β), intracellular Ca^2+^ mobilization and integrin αIIbβ3-mediated “outside-in” signal transduction (inhibiting the phosphorylation of src). (Inhibit 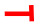; Induce 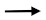)

The effects of sanguinarine on arterial thrombosis in vivo and in vitro were investigated in this study. Sanguinarine dose-dependently inhibited human platelet aggregation, α-granule and dense granule release, αIIbβ3 activation and intracellular calcium mobilization induced by collagen. It also inhibited platelet spreading on immobilized fibrinogen. This suggests that sanguinarine inhibits collagen-induced platelet activation as well as integrin αIIbβ3 “outside-in” signal transduction. From the molecular mechanism, it was clear that sanguinarine inhibited the phosphorylation of downstream signal molecules of the collagen specific receptor GPVI signal pathway, including Syk-PLCγ2 and PI3K-Akt-GSK3β. Moreover, it also inhibited αIIbβ3-mediated β3-Src signaling (Figure 8). Sanguinarine inhibited artery thrombosis induced by FeCl_3_ in mice, but had no influence on tail bleeding time. This suggests that it had no obvious risk of bleeding.

Platelet adhesion and aggregation is crucial in the process of arterial thrombosis [1,27]. A recognized arterial thrombosis model is FeCl_3_-induced carotid artery thrombosis, in which multiple anticoagulants and antiplatelet drugs are sensitive to [28]. Therefore, sanguinarine has well-established antithrombotic properties in mice by prolonging the closure time of blood vessels while not increasing the tail bleeding time (Figure 2a,b). In vivo, the shear force exerted by blood laminar flow is considered an important environmental factor in the process of platelet activation under normal and pathological conditions [2]. It was reported that the shear rates of 1002.6 s^−1^, 2282.67 s^−1^ and 3989.248 s^−1^ represent shear conditions in normal arteries, moderately stenotic arteries and severely stenotic arteries, respectively [21]. To check sanguinarine antiarterial thrombosis in vitro, we used Bioflux 1000Z to analysis human blood thrombus formation in high shear force (2000 s^−1^) simulating arterial stenosis. The results presented that sanguinarine significantly reduced the adhesion area (Figure 2c,d) of platelets in flow, which suggested the possibility of antithrombosis in clinic.

In Jeng’s study [14], the concentration of collagen used in platelet aggregation was 10 μg/mL, which was much higher than that in physiological and pathological conditions. In our study, we used 1 μg/mL collagen and 0.25 μg/mL collagen-related peptide to activate platelets. As shown in Figure 3a,b, sanguinarine revealed significant antiaggregation in a dose-dependent manner. Three types of platelet secretory granules (α granules, dense granules and lysozyme) release different contents when platelets are activated to participate in various pathophysiological processes such as hemostasis, thrombosis and immune reaction [29]. Platelet α granules are rich in P-selectin, fibrinogen and vWF, and detection of the expression of P-selectin on the membrane reflects α granule release during platelet activation. While dense granules contain ADP, ATP and 5-HT, detection of ATP reflects dense granule release [30]. As shown in Figure 4, sanguinarine significantly inhibited ATP release and the expression of P-selectin, which suggests that sanguinarine inhibits both α granule and dense granule release. In addition to P-selectin expression, the direct binding of monoclonal antibody PAC-1 is considered to be another key marker of platelet activation [31]. Sanguinarine decreased collagen-induced platelet αIIbβ3 activation in a dose-dependent manner, which further confirmed its inhibitory effect on platelet “inside-out” signal transduction (Figure 4e,f).

Collagen activates signal transduction based on Src family kinase (SFK) by binding to GPVI receptors, resulting in activation of Syk/PLCγ2/PKC [32,33]. Through the analysis of precipitates after collagen-induced platelet aggregation, it was likely that sanguinarine reduced the Syk phosphorylation more than that of PLCγ2. PI3K is an earlier-reported signal subunit [34,35] that is also activated by collagen-GPVI mediated PLCγ2. A serine/threonine kinase, AKT, which is the main downstream effector of PI3K, is attracted to the platelet plasma membrane through its PIP3 binding domain and then phosphorylated by PDK1 and mTORC2 [23]. The study conducted by Barry [3] has shown that AKT has an important role in collage-induced platelet activation via the GPVI receptor. In addition, the role of GSK3β in platelet function was also proved by inhibitors and genetic experiments [24]. Our results confirmed that sanguinarine also acted as a repressor of PI3K-Akt-GSK3β signaling to weaken collagen-stimulated platelet activation.

The increase of intracellular Ca^2+^ in platelets is a key signal event that is essential for most of the major functional responses in the process of platelet activation, including cytoskeleton rearrangement, particle release and integrin αIIbβ3 inside-out signaling [22,36]. A study by Rink et al. confirmed that in the process of activating platelets, various agonists, such as collagen, TxA2, ADP and thrombin, lead to an increase concentration of intracellular Ca^2+^, although they have an effect on different platelet receptors and activate different signal pathways [25]. The key second messenger downstream of most platelet signaling pathways is Ca^2+^, so the regulation of Ca^2+^ signal could be an interesting target for platelet inhibition [37]. Our study indicated that sanguinarine greatly inhibited the increase of [Ca^2+^]i in platelets induced by collagen, and the dose-dependent trend was consistent with its antiplatelet aggregation effect (Figure 6a,b).

Fibrinogen plays a crucial role in cytoskeleton reorganization and platelet spreading after stabilizing thrombosis and growth by binding to the activated integrin αIIbβ3 and triggering the “outside-in” signal [26,38]. At the beginning of the outside-in signal, integrin αIIbβ3 subunit β3 can be phosphorylated, resulting the phosphorylation of Src (Tyr416), which then causes the phosphorylation of other downstream proteins [39]. Our study confirmed that sanguinarine inhibited the phosphorylation of β3 and Src in spreading platelets on fibrinogen, which indicated that it had a significant inhibitory effect on αIIbβ3-mediated outside-in signaling.

However, both previous studies [14] and our results have confirmed that sanguinarine can also inhibit thrombin-induced platelet aggregation (data not shown). Whether sanguinarine inhibits the platelet function by negatively regulating thrombin receptor-linked signaling pathways will be confirmed in future experiments.

## 5. Conclusions

Our study indicated that sanguinarine inhibited arterial thrombosis in a dose-dependent manner. Additionally, sanguinarine inhibited platelet aggregation, granule release and spreading. The underlying mechanisms are as follows (Figure 8): 1. Inhibiting the downstream signal molecules of the collagen-specific GPVI receptor signal pathway, including the phosphorylation of Syk-PLCγ2 and PI3K-Akt-GSK3β; 2. Inhibiting collagen-induced intracellular calcium mobilization; 3. Negatively regulating the αIIbβ3 “outside-in” signal transduction. The study suggests that sanguinarine may be a potential candidate for the treatment of thrombotic disease.
